# Trajectories of emotional and behavioral problems in young children during the COVID-19 pandemic: A longitudinal study

**DOI:** 10.1371/journal.pmen.0000265

**Published:** 2025-02-27

**Authors:** Markus A. Landolt, Noëmi Ruther, Nathan L. Strebel, Stefanie J. Schmidt

**Affiliations:** 1 Department of Psychosomatics and Psychiatry, Children’s Research Center, University Children`s Hospital Zurich, University of Zurich, Zurich, Switzerland; 2 Division of Child and Adolescent Health Psychology, Department of Psychology, University of Zurich, Zurich, Switzerland; 3 Division of Clinical Child and Adolescent Psychology, University of Bern, Bern, Switzerland; University of Lisbon: Universidade de Lisboa, PORTUGAL

## Abstract

The COVID-19 pandemic has had profound effects on mental health of children. This study aimed to explore the trajectories of emotional and behavioral problems in children aged 1–6 years over four time points from April 2020 to May 2021 and to identify predictors of these trajectories. This longitudinal study involved four assessments with anonymous online surveys completed by 527 - 888 parents of children aged 1–6 years in Austria, Germany, and Switzerland. Emotional and behavioral problems were measured using the Child Behavior Checklist (CBCL/1.5-5). Sociodemographic data, COVID-19 exposure, child worries, and parental mental health were also assessed. The prevalence of clinically significant behavioral problems ranged from 4.2% to 11.5%, higher than the normative 2%. Latent class growth analyses identified two trajectory classes for anxiety and affective problems: low-symptom and high-symptom classes. For oppositional-defiant problems, three classes emerged: low, medium, and high-symptom classes. The high-symptom classes constituted between 13.1% and 15.6% of the sample, depending on the symptom domain. High symptom trajectories showed little change over time and were significantly predicted by parental mental health (anxiety and depression; OR 1.13–1.21) and child worries (OR 1.19–1.24), with female sex being a predictor for high affective problems (OR 1.90). This study shows that a significant minority of young children experienced elevated emotional and behavioral problems during the first year of the pandemic, primarily influenced by parental mental health and child worries. The identification of a relatively stable high-symptom class points to the need for targeted and timely interventions for those at higher risk. These findings emphasize the importance of supporting parental mental health and addressing children’s worries to mitigate the adverse effects of the pandemic on young children’s mental health.

## Introduction

The global COVID-19 pandemic emerged in late 2019 and prompted widespread global responses, including lockdowns, social distancing measures, face mask mandates, travel restrictions, and vaccination campaigns [1] in an effort to control the spread of the virus and mitigate its impact on healthcare systems. The pandemic had far-reaching effects on public health, the economy, education, and social interactions, resulting in significant challenges for individuals, communities, and governments worldwide. Numerous studies have demonstrated the pandemic’s negative effects on the mental health of school-aged children, adolescents, and adults [2–6]. In their overview of systematic reviews, Harrison et al. [7] found a pooled prevalence of 32% for depression (95% CI: 27 to 38, n=161,673) and 32% for anxiety (95% CI: 27 to 37, n=143,928) in children and adolescents globally. Most studies focused on older children and adolescents, some including mixed samples with younger children [8]. However, there is still little research among young children below the age of 6 years, despite the pandemic significantly affecting their daily lives too, and although they might be a particularly vulnerable group due to possible difficulties in understanding the pandemic and its associated measures [9]. An interesting qualitative study by Vasileva et al., 2021 [10] indicated that children aged 3 to 5 years often overestimated the risk of a COVID-19 infection. Caregivers reported that their children expressed worries about falling ill, transmitting the illness to others, and the permanence of changes in daily life. Children were reported to be preoccupied with COVID-19 and their worries were evident in conversations, playtime activities, and drawings.

It is therefore not surprising, that available, mainly cross-sectional studies among preschoolers have indicated increased psychological distress and negative mood [11–14], anxiety [15, 16], affective symptoms [12,15] along with an increase in externalizing problems, such as hyperactivity [12,13,17,18], aggression [17], and oppositional-defiant behavior problems [15,19]. Furthermore, studies by Delvecchio et al. [20], Alonso-Martinez et al. [21] and Orgilés et al. [6] reported an increase in sleep problems among young children.

Although cross-sectional studies do not allow to draw causal conclusions, several socio-demographic, individual, pandemic-related, and parental risk factors for poor mental health and behavior problems in young children have been identified. Special educational needs and preexisting health problems of the child [13], disruption of family routines [12], lower socioeconomic status [13,22], longer duration of quarantine [23], and parental divorce [13] were found to be related to an increase in child symptoms. Additionally, a strong and consistent association has been found between parental distress and mental health and child emotional and behavioral problems (e.g., [15,24–27], notably also in a prospective study with three assessments between April and June 2020 [28]).

While some insights into the psychological repercussions of the pandemic on young children have emerged, several noteworthy limitations remain unaddressed. First, previous studies in this age group are mainly cross-sectional, which limits the ability to infer causal relationships between variables. Cross-sectional designs capture data at a single point in time, making it difficult to determine the directionality of associations or whether observed relationships are influenced by underlying confounding factors. Consequently, longitudinal studies are needed to track developmental trajectories and better understand how certain factors contribute to outcomes over time. Second, there is limited research on how symptoms in preschoolers evolved throughout the pandemic, particularly concerning the identification of trajectory groups and associated sex differences in symptomatology. Third, research on the effects of specific pandemic-related exposures (e.g., having ill family members, having lost a loved one to COVID-19) is also sparse. Finally, there is little information on the specificity of risk factors for internalizing and externalizing problems. More specifically, it is unclear whether the same risk factors are relevant for different emotional and behavioral problems.

To address some of these limitations, the present study aimed at examining the following three research questions: (1) How did the mental health and behavioral patterns of young children evolve from April 2020 to May 2021? (2) Can different trajectories of mental health and behavior problems be identified during the study period? (3) Can these trajectories be predicted by sociodemographic, parental, and pandemic-related factors?

## Method

### Ethics statement

The study received ethical approval by the Institutional Review Boards of the universities of Zurich (#20.4.1) and Bern (#2020-04-00002). Participants provided written informed consent.

### Participants and study design

This longitudinal study comprised four anonymous online surveys lasting approximately 15–20 minutes each. The study assessed three age groups using different methods (1–6 years, 7–10 years, and 11–19 years). The results of the first wave have been published previously [15]. The current paper reports on the longitudinal data of the youngest cohort, comprising children aged 1–6 years.

**Fig 1:**
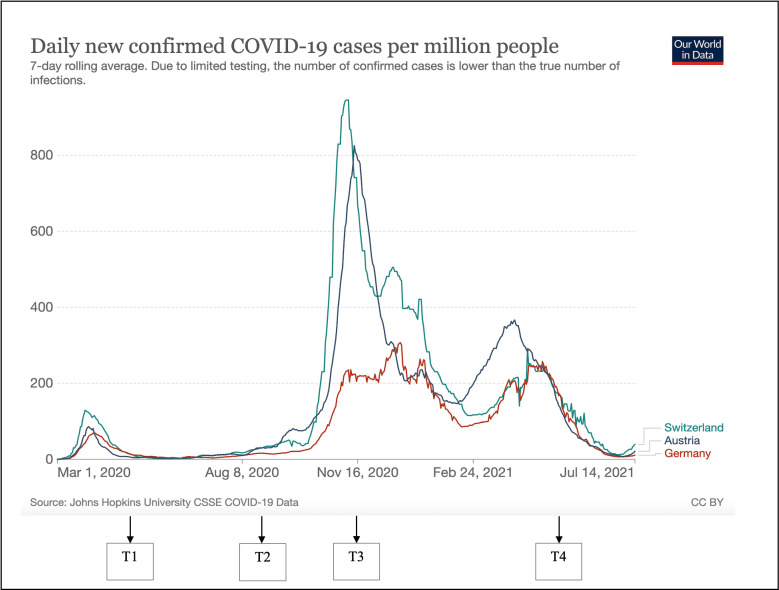
Daily new confirmed COVID-19 cases per million people in Switzerland, Germany, and Austria during the study period.

Participants were eligible if they (1) were residents in Austria, Germany, or Switzerland (incl. Liechtenstein); (2) were parents/caregivers of a child aged between 1 and 6 years at the first assessment; and (3) had sufficient German language skills to understand the questions. Participants were assessed four times: T1, between April 9 and May 11, 2020 (N=2726); T2, between August 3 and August 31, 2020 (N=664); T3, between November 3 and December 2, 2020 (N=527); and T4, between May 3 and May 31, 2021 (N=608). Fig 1 illustrates the number of new COVID-19 cases in Austria, Germany, and Switzerland during the study period. T1 of our study corresponded to a phase marked by significant and largely synchronized lockdown measures in these countries, including the closure of day-care facilities and kindergartens. The assessment at T2 was undertaken during a period with low numbers of new cases and relatively few restrictions in all three countries. In contrast, T3 coincided with the peak of the pandemic’s most severe wave. The final assessment at T4 was shortly after the fourth, less severe wave of the pandemic in these three countries. The predominant COVID-19 virus variant in all three countries from T1 to T3 was the pre-Alpha variant (wild type), with T4 marking the onset of the Alpha variant (B.1.1.7) dominance [29,30]. Compared to the general population, young children showed noticeably lower incidences of COVID-19 infection at each measurement point [31–34]. For example, in Switzerland, the incidence rates at T1 were 1.6 per 100,000 inhabitants for young children compared to 13.6 for the total population, increasing by T3 to 32.9, and 326.6, respectively [31].

Recruitment of participants was performed via social media, e-mail circulation, websites, newspapers, and magazines. Parents were provided with a link to the online survey which included confirmation of informed consent on the first page. Parents of children aged 1–6 years participating in the first study wave were asked to provide their email addresses if they were willing to be re-contacted to participate in the following waves. These parents then received the link to the follow-up surveys (T2-T4). Analyses of dropouts between T1 and the following assessments revealed that parents with higher levels of depression and anxiety less frequently participated in the T2 and T3 assessments (p<.05). Additionally, parents with lower education were underrepresented in the fourth assessment (p=.02). The survey was predominantly completed by mothers (T1: 88.3%; T2: 90.2%; T3: 91.7%; T4: 90.6%).

### Measures

#### Child emotional and behavioral problems.

Emotional and behavioral problems were assessed by the following three DSM-oriented subscales from the German version of the parent-reported Child Behavior Checklist for ages 1.5–5 years (CBCL/1.5–5; [35]) affective problems (10 items), anxiety (10 items), and oppositional-defiant behaviors (6 items). This instrument is widely used across the globe, well-validated, and provides cut-off scores for clinical and borderline clinical problems [36].

#### Predictors.

The following covariates were assessed: sociodemographic variables (child age, child sex, parental education), exposure to COVID-19, COVID-19-related child worries, and parental mental health.

*Parental education* was assessed at T1 by asking for the highest level of education attained by the mother and the father. Five response options were available, ranging from 1 (mandatory school not completed) to 5 (university). A sum score of parental education was calculated by adding the scores of maternal and paternal education.

*Exposure to COVID-19* was measured through self-generated items concerning events related to COVID-19. These events encompassed the following 7 exposures: child had COVID-19, sibling’s diagnosis of COVID-19, father’s diagnosis of COVID-19, mother’s diagnosis of COVID-19, family member’s quarantine due to COVID-19, family member’s hospitalization due to COVID-19, and loss of loved one due to COVID-19. A sum score was computed by assigning a score of 1 for each confirmed item and a score of 0 for non-occurrence. At each assessment point, this scale was applied to the corresponding interval between surveys. A composite score across all time points was computed, representing the overall extent of COVID-19 exposure spanning from April 2020 to May 2021 (range 0–28).

The *child’s COVID-19-related worries*, as reported by the parents, were assessed with two self-generated items: “Is your child worried about getting sick or dying?” and “Is your child worried that a family member might get sick or die?” Three response options were available (0 = not at all; 1 = somewhat; 2 = very much). Child worries were assessed at all four time points. For analyses, a sum score was calculated across all time points, with a higher score representing more worries (range 0–16).

*Parental mental health* was evaluated across all time points using the Patient Health Questionnaire-9 (PHQ-9; [37]) and the General Anxiety Disorder-7 scale (GAD-7; [38]). These well-established screening tools [39,40] were employed to gauge the level of depressiveness and anxiety over the preceding two weeks. The PHQ-9 is a 9-item self-report questionnaire that evaluates the severity of depressive symptoms based on DSM-IV criteria for major depression. The GAD-7 is a 7-item self-report questionnaire designed to assess the severity of generalized anxiety disorder symptoms over the past two weeks. In both, each item is scored on a 4-point Likert scale (0 = Not at all to 3 = Nearly every day). Clinically significant symptomatology was determined based on established cutoffs. In the present study, the sum score of each measure was used to describe the degree of mental health impairment at each time point. Additionally, a mean score over all time points was calculated for both scales separately, providing an overall representation of mental health during the study period.

### Statistical analyses

Individual data from T1 to T4 were linked according to the provided email addresses. For the current analyses, 888 participants who completed at least two of the four surveys, were included. Data were analyzed using SPSS, version 29 (IBM Inc.), and Mplus 8, version 1.7 [41]. All *p*-values below 0.05 were considered statistically significant. We calculated means and standard deviations for continuous variables and frequencies for categorical variables.

For our first aim, latent class growth analysis (LCGA), assuming no inter-individual changes in within-class variation [41], was used to identify different homogenous subpopulations with comparable growth trajectories over time (‘latent classes’). LCGA postulates homogeneity within classes and variance and covariance estimates for growth factors (i.e., intercept and slope) are therefore fixed to zero. LCGA was performed using maximum likelihood estimation in Mplus with the three CBCL-subscales raw data of all four assessment points to delineate subgroups characterized by distinct trajectories. For each class *c*, the trajectory of individual *i* at time *t* is modeled as


$$Y_{it}=\eta_{0c}+\eta_{1c}\cdot t+\varepsilon_{it}$$

Unconditional models were established with varying numbers of class solutions, extending up to five classes. We estimated linear and quadratic effects. Model selection criteria included lower values of the Bayesian Information Criterion (BIC), sample-size adjusted BIC (nBIC), Akaike Information Criterion (AIC) compared to other models, higher entropy values, and statistically significant Lo–Mendell–Rubin and bootstrap likelihood ratio tests (LMR-LRT). In addition to these criteria, minimal sample size of classes was considered to ensure sufficient power for subsequent statistical analyses.

To examine the factors predicting the higher symptom class trajectories, we conducted three logistic regression analyses using SPPS based on the following theoretical formula:


$$\log\left(\frac{p_{i}}{1-p_{i}}\right)={\text{β}}_{0}+{\text{β}}_{1}X_{i1}+{\text{β}}_{2}X_{i2}+{\text{β}}_{3}X_{i3}+{\text{β}}_{4}X_{i4}+{\text{β}}_{5}X_{i5}+{\text{β}}_{6}X_{i6}+{\text{β}}_{7}X_{i7}$$

Any missing data were addressed through multiple imputations, under the assumption that the data were missing at random [42]. The classes derived from the LCGA were entered as dependent variables into the regression models. Selection of predictors was based on their significance in our cross-sectional findings at T1 [15] and the existing literature among young children. The suitability of variables for the regression analyses was assessed by examining their distribution, homoscedasticity, outliers, and multicollinearity. Regarding distribution, we observed that the predictor variables and residuals were not normally distributed across all levels of the independent variables. In addition, our data showed clear absence of homoscedasticity. However, as normality and homoscedasticity are not requirements for a logistic regression, this is unlikely to have influenced our findings [43]. Regarding outliers, none of the predictors, except for GAD and PHQ, exhibited more than three outliers (i.e., 0.33% of the sample). The most critical requirement for logistic regression analysis is the absence of multicollinearity. In this study, all variables demonstrated low to moderate multicollinearity (VIF < 5), with moderate multicollinearity observed only for the PHQ and GAD variables.

## Results

### Sample characteristics

Table 1 provides an overview of the sample characteristics. At the first assessment, 45.5% of the participants were from Switzerland/Liechtenstein, 37.8% from Germany, and 16.7% from Austria. Almost all children were living with two parents (95.3%). Twelve children (1.4%) were in psychological treatment before the pandemic began, while 31 children (3.5%) had a pre-existing chronic physical condition (e.g., asthma). Around half of the parents had a university education (55.7% of mothers; 47.6% of fathers). Across the four assessment points, the proportion of children exceeding the clinical cutoff ranged from 4.2% to 5.9% for anxiety, from 5.1% to 8.6% for affective problems, and from 6.0% to 11.5% for oppositional-defiant problems (Table 1).

**Table 1:** Sample characteristics across the four assessments.

	T1	T2	T3	T4
N	888	664	527	608
Child age, M (SD)	3.79 (1.32)	4.07 (1.34)	4.30 (1.35)	4.81 (1.37)
Child female sex, n (%)	455 (51.24)	339 (51.05)	267 (50.66)	320 (52.63)
Child exposure to COVID, n (%)				
Child had COVID	5 (0.6)	1 (0.2)	5 (0.9)	39 (6.4)
Sibling had COVID	3 (0.3)	2 (0.3)	3 (0.6)	35 (5.8)
Mother had COVID	7 (0.8)	2 (0.3)	17 (3.2)	29 (4.8)
Father had COVID	6 (0.7)	2 (0.3)	15 (2.8)	32 (5.3)
Family member in quarantine	67 (7.5)	38 (5.7)	113 (21.4)	163 (26.8)
Family member in hospital due to COVID	2 (0.2)	2 (0.3)	3 (0.6)	5 (0.8)
Death of someone due to COVID	3 (0.3)	4 (0.6)	3 (0.6)	17 (2.8)
COVID exposure score M (SD)	0.11 (0.41)	0.08 (0.32)	0.30 (0.71)	0.53 (1.02)
Child worries, M (SD)	0.65 (0.89)	0.65 (0.91)	0.72 (0.91)	0.82 (0.93)
n (%) ≥ 2	165 (18.6)	145 (21.8)	123 (23.3)	174 (28.6)
CBCL Anxiety, M (SD)	2.91 (2.64)	2.86 (2.75)	3.03 (2.66)	3.17 (2.94)
n (%) above cutoff^1^	37 (4.2)	32 (4.8)	29 (5.5)	36 (5.9)
CBCL Affective, M (SD)	2.38 (2.54)	2.20 (2.23)	2.20 (2.37)	2.56 (2.65)
n (%) above cutoff^1^	76 (8.6)	39 (5.9)	27 (5.1)	50 (8.2)
CBCL Oppositional-				
Defiant, M (SD)	4.20 (3.04)	4.05 (2.71)	4.22 (2.85)	4.45 (3.01)
n (%) above cutoff^1^	80 (9.0)	40 (6.0)	42 (8.0)	70 (11.5)
Parental PHQ-9, M (SD)	5.21 (4.00)	4.06 (3.87)	4.80 (3.92)	5.71 (4.53)
n (%) above cutoff^2^	122 (13.7)	59 (8.9)	62 (11.8)	105 (17.3)
Parental GAD-7, M (SD)	4.43 (3.56)	3.49 (3.47)	4.00 (3.57)	4.64 (3.87)
n (%) above cutoff^3^	87 (9.8)	40 (6.0)	45 (8.5)	69 (11.4)

^1^Cutoff for clinical problems (T≥70) based on manual [35].

^2^Cutoffs for moderate to severe depression based on the manual [37].

^3^Cutoffs for moderate to severe anxiety based on the manual [38].

### Identifying trajectories

To detect subgroups characterized by distinct trajectories in anxiety, affective problems, and oppositional-defiant problems we first ran latent class growth models without covariates (unconditional models) with up to five classes. Fit indices for each of the three measures for all classes are presented in Table 2.

**Table 2:** Fit indices for latent class growth models for anxiety, affective problems, and oppositional-defiant problems.

Class	AIC	BIC	Sample-size adjusted BIC	Entropy	p-value for LRM-LRT	Smallest class N (%)
*Anxiety*						
1	13042	13075	13053	1	n/a	n/a
**2**	**12191**	**12243**	**12208**	**0.90**	**.002**	**139 (15.4)**
3	11921	11993	11944	0.86	.07	37 (4.11)
4	11839	11931	11870	0.79	.17	19 (2.11)
5	11783	11893	11820	0.81	.56	15 (1.67)
*Affective problems*						
1	12445	12479	12457	1	n/a	n/a
**2**	**11463**	**11515**	**11480**	**0.91**	**<.001**	**130 (14.4)**
3	11143	11215	11167	0.87	.01	43 (4.8)
4	11041	11132	11072	0.85	.38	42 (4.7)
5	10929	11039	10966	0.82	.18	26 (2.9)
*Oppositional-defiant problems*						
1	13378	13412	13390	1	n/a	n/a
2	12680	12733	12698	0.73	<.001	318 (35.3)
**3**	**12442**	**12514**	**12466**	**0.73**	**<.001**	**110 (12.2)**
4	12386	12477	12417	0.71	.01	37 (4.1)
5	12323	12434	12361	0.76	.008	34 (3.8)

AIC = Akaike information criterion; BIC = Bayesian information criterion; LMR-LRT = Lo-Mendel-Rubin likelihood ratio test (adjusted).

**Fig 2:**
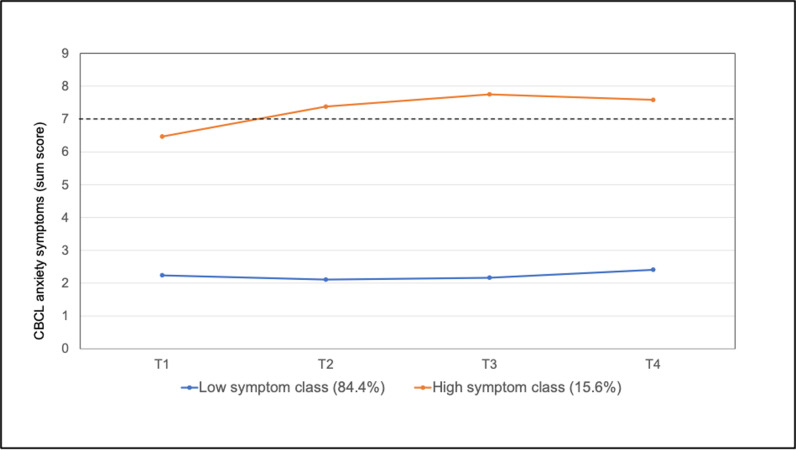
Estimated trajectories of anxiety symptoms by latent classes (dotted line represents the cutoff for borderline clinical symptomatology).

For anxiety problems, a two-class solution was identified as the most appropriate model to account for differences in trajectories. This solution was supported by a significant p-value for the LRM-LRT (p = 0.002) and a satisfactory sample size in the smaller class (n = 139, 15.4%). Additionally, the 2-class solution demonstrated a higher entropy-value than the 3-class solution. The estimated trajectories for anxiety problems are shown in Fig 2, and the statistical parameters can be found in S1 Table. We characterized the two classes as a low-symptom class and a high-symptom class (with symptom levels from T2 to T4 consistently above the borderline clinical cutoff). The low-symptom class showed a significant quadratic change over time (p = 0.03), while the linear slopes for both classes did not reveal significant changes over time. Descriptive analyses of the slopes indicate a slight increase in symptom severity in the high symptom class, with the highest anxiety expression at T3. For the low symptom class, a small decrease in anxiety can be observed, before symptoms rise again from T3 to T4.

**Fig 3:**
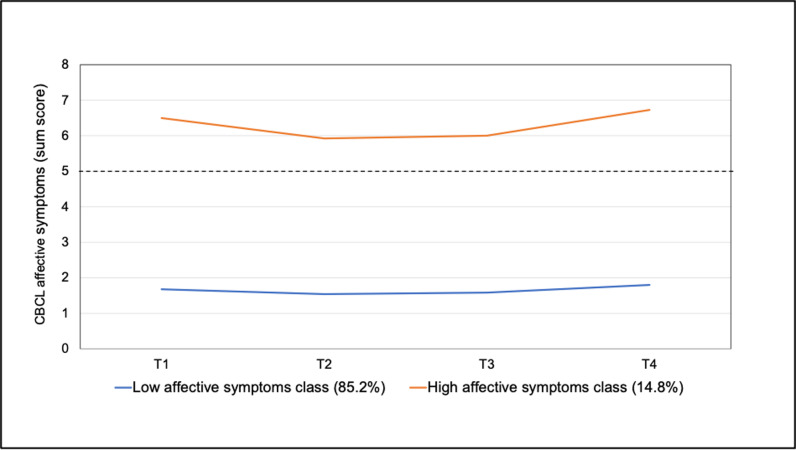
Estimated trajectories of affective symptoms by latent classes (dotted line represents the cutoff for borderline clinical symptomatology).

Regarding affective problems, a two-class solution was determined to be the best fit for the data (Table 2). While a three-class solution also yielded a significant p-value for the LRM-LRT, the two-class solution was preferred due to its higher entropy value (0.91) and a larger number of participants in the smallest class (n = 130, 14.4%). The estimated trajectories for the two classes related to affective problems are illustrated in Fig 3, with detailed parameters provided in S1 Table.

Similar to the trajectory classes for anxiety symptoms, the two classes were identified as a low-symptom class and a high-symptom class (consistently above the borderline clinical cutoff). Both the low-symptom class (p = 0.006) and the high-symptom class (p = 0.03) showed significant quadratic changes over time. Notably, a significant negative slope was observed only in the low-symptom class (p = 0.03). The high-symptom class demonstrated a substantial dip in affective problems at T2 (negative slope) but showed an increase again at T4. The low-symptom class reported fewer symptoms over time, with the rate of change decreasing (negative slope and positive quadratic term).

**Fig 4:**
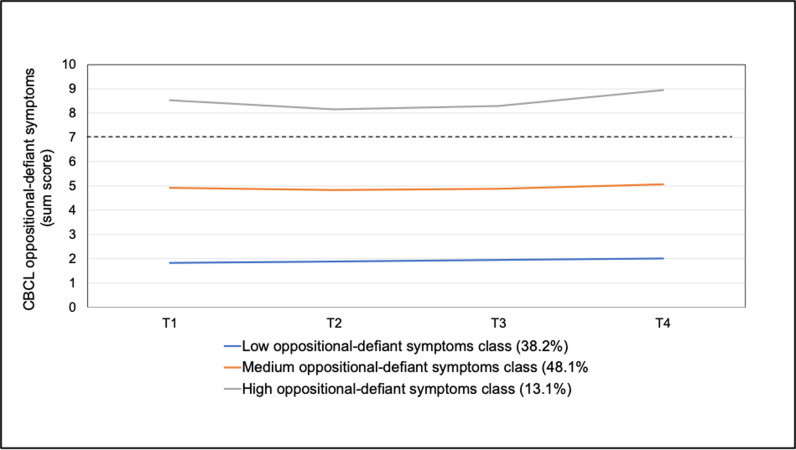
Estimated trajectories of oppositional-defiant symptoms by latent classes (dotted line represents the cutoff for borderline clinical symptomatology).

For oppositional-defiant problems, all class solutions resulted in a significant LRM-LRT (Table 2). The three-class solution was selected due to having the most satisfying number of participants in the smallest class (n = 110, 12.2%) and better AIC and BIC values compared to a two-class solution. The classes were characterized as low, medium and high symptom classes, with the latter being consistently above the clinical cutoff across all time points. The trajectories of the three classes are provided in Fig 4. As indicated in S1 Table, neither the slope nor the quadratic term was significant for any of the classes, suggesting relative stability in trajectories over time. However, the high symptom class initially shows a decrease in symptomatology (negative slope), before rising slightly above the original level at T4 again.

### Predictors of trajectory class

To establish predictors of higher symptom trajectory classes, we conducted logistic regression analyses, the results of which are shown in Table 3.

**Table 3:** Logistic regression analysis predicting membership in high symptoms trajectory class for anxiety, affective and oppositional-defiant symptoms.

Anxiety						Affective					Oppositional-defiant				
*Predictor*	*B*	*SE*	*OR*	*95% CI*	*p*	*B*	*SE*	*OR*	*95% CI*	*p*	*B*	*SE*	*OR*	*95% CI*	*p*
Exposure to COVID-19 (composite score)	0.09	0.09	1.10	0.92–1.31	.29	-0.09	0.11	0.91	0.73–1.14	.41	-0.12	0.10	0.88	0.73–1.08	.22
Female sex	0.06	0.21	1.06	0.70–1.61	.78	0.64	0.23	1.90	1.21–2.98	.005	-0.13	0.23	0.88	0.56–1.38	.58
Child age	-0.15	0.09	0.86	0.72–1.03	.10	-0.15	0.10	0.86	0.71–1.04	.11	0.13	0.10	1.14	0.94–1.38	.18
Parental education	0.05	0.08	1.05	0.90–1.23	.55	-0.08	0.08	0.93	0.79–1.09	.35	-0.09	0.08	0.91	0.78–1.08	.27
Mean parental anxiety	0.15	0.06	1.16	1.04–1.31	.010	0.09	0.06	1.09	0.98–1.22	.12	0.14	0.06	1.15	1.03–1.29	.016
Mean parental depression	0.12	0.05	1.13	1.02–1.25	.015	0.24	0.05	1.27	1.15–1.41	<.001	0.19	0.05	1.21	1.09–1.34	<.001
Child worries	0.21	0.04	1.24	1.15–1.34	<.001	0.17	0.04	1.19	1.09–1.29	<.001	0.05	0.04	1.05	0.97–1.15	.23

*N* = 888. B = Regression coefficient; SE = Standard error of regression coefficient; OR = Odds Ratio; CI = Confidence interval; p = pooled p-value across all five imputed datasets.

As displayed in Table 3, membership in high anxiety trajectory class was significantly predicted by parental anxiety, parental depression, and child worries. Among these predictors, child worries emerged as the strongest, with an odds ratio of 1.24.

Similar to the findings for anxiety, significant predictors for membership in the high affective problems class include parental depression and child worries. Additionally, female children were nearly twice as likely to be classified into the high affective problems group compared to male children.

Regarding oppositional-defiant symptoms, parental anxiety, and parental depression emerged as significant predictors. Notably, parental depression was the most predictive factor, with children of parents exhibiting high depression scores being 1.21 times more likely to fall into the high oppositional-defiant symptoms group.

## Discussion

The present study aimed at examining how the mental health and behavioral patterns of young children evolved from April 2020 to May 2021. Specifically, our goal was to identify different trajectories of change and their predictors.

### Outcomes across time points

Overall, across the four assessments, the proportion of young children in our sample with clinically significant problems ranged from 4.2% to 11.5%, which is substantially higher than the expected 2% based on normative data [44], but lower than in other studies (e.g., [12]). Regarding the type of behavioral problems, we found both elevated internalizing (affective and depressive) and externalizing (oppositional) symptoms which is in line with two cross-sectional studies from Canada and Japan [45,46]. Interestingly, the proportion of children with clinically significant anxiety symptoms slightly increased over time, whereas the proportion of children with clinically significant oppositional behavior problems and depressive symptoms fluctuated. Notably, fewer children exceeded the clinical cut-off at T2 and T3. Other studies (involving adolescents or adults) also reported a decrease in symptoms several months after the onset of the pandemic [47,48], although this trend was not observed for all symptom domains in children [49]. Our results can probably in part be explained by changes in the epidemiological situation, as, for example, relatively few infection cases were recorded during the second survey (August 2020). Thus, many of the stringent measures had been lifted (see Fig 4). The fourth survey, in contrast, was conducted after the highest peak of cases. The fluctuating pandemic with distinct waves of infections and control measures was likely associated with different levels of stress for the families. The high and fluctuating rates of parent mental health problems across the four assessments in this study support this hypothesis.

### Trajectory classes

One of the primary objectives of this study was to identify distinct trajectory classes for various symptom domains. For anxiety and affective problems, two classes were identified: a low-symptom class and a high-symptom class. In contrast, for oppositional-defiant problems, a third, medium-symptom class emerged. The high-symptom class constituted between 13.1% and 15.6% of the sample, depending on the symptom domain. This highlights the resilience of the vast majority of young children. Across all classes, trajectories were relatively stable. Initial symptoms appeared crucial for the subsequent course, as children exhibiting higher symptoms early on tended to maintain them throughout the pandemic, while resilient children remained stable in their responses over time.

Our findings partially align with Raw et al. [49], who also found resilient and chronic classes during the COVID-19 pandemic among British children and adolescents. However, Raw et al. [49] identified additional classes with more significant changes over time. Similarly, Guzman-Holst et al. [26] studied 3322 UK children and adolescents aged 4–16 and their symptom trajectories associated with COVID-19. They too, identified five trajectories for emotional problems, four for conduct problems, and four for hyperactivity/inattention. Most children and adolescents showed low stable symptoms, but a significant proportion did experience moderate to high stable symptoms. However, except for the high to moderate (decreasing) trajectory group in emotional problems, none of the unstable groups encompassed more than 7% of the sample. A similar result is reported by Kaman et al. [50] who also found five trajectory classes for externalizing mental health problems and four for internalizing mental health problems. They too report that the largest amount of the sample remained stable at a low internalizing and externalizing level, highlighting the importance of initial symptom levels for trajectory over the subsequent course. Possible explanations for these three studies finding more classes are their larger sample sizes and the wider age range of their participants. As Schmidt et al. [15] have shown, there are considerable age differences with regard to the emotional and behavioral problems children and adolescents developed after the onset of the pandemic, which again increases the possibility of finding more different trajectory classes.

### Predictors of class membership

The third aim of the present study was the analysis of sociodemographic, parental, and pandemic-related predictors of trajectory classes. Regarding pandemic-related factors, we found that COVID-19 exposure did not predict class membership with regard to all outcomes. Our results align with previous studies that reported similar findings [13,51–53]. Several factors could explain this finding. First, the overall low exposure scores of our participants may have contributed to this lack of significance. Additionally, our sample consisted predominantly of individuals with higher education, which may have mitigated the impact of COVID-19 exposure on psychological outcomes. This possible explanation is in accordance with a study by Rajmil et al. [54], who found an increase in social inequalities and that in high-income countries, the most vulnerable children faced greater negative impacts on mental and physical health, along with increased rates of child abuse and maltreatment. In the same vein, Ravens-Sieberer et al. [55] conclude that socially disadvantaged children and adolescents are at a particularly high risk of experiencing low health-related quality of life, mental health problems, and depressive symptoms during the pandemic. Finally, Villaume et al. [56] found that adolescents from low/moderate education households experienced heightened stress and unique mood changes (more shame, care, and excitement) during the pandemic, compared to their high-education peers. While mood shifts diminished over time, home- and health-related stress remained elevated.

While child age was not a predictor of symptoms (probably due to the limited age range of our participants), child sex significantly predicted affective symptoms, with girls almost twice as likely to be classified into the high affective problems group compared to boys. This finding aligns with Raw et al. [49] and broader literature indicating females to be prone to internalizing symptoms, typically emerging during adolescence [57]. Our results suggest female children might react more strongly to stressors with internalizing symptoms. No significant differences were found between boys and girls regarding oppositional-defiant symptomatology, though there was a tendency toward higher prevalence in boys. Notably, the first wave of data collection in our study also revealed an increase in oppositional behavior problems more frequently among male children [15].

This study also examined parental psychopathology as a predictor of class membership. Parental anxiety emerged as a significant predictor for both anxiety and oppositional-defiant problems in their offspring. The proportion of parents experiencing moderate to severe anxiety symptoms varied from 6.1% to 11.4%, depending on the assessment time point. This variation highlights the fluctuating nature of anxiety levels in response to the evolving pandemic situation. This association between parental and child psychological problems is well-documented in the epidemiological and pandemic literature [5,12,13,22,25,28,46,49,58–62].

Parental depression was identified as the sole predictor consistently accounting for differences in group membership across all examined child symptoms: affective problems, anxiety, and oppositional-defiant behaviors. The prevalence of moderate to severe depression symptoms among parents ranged from 2.2% to 6.1%, which is notably lower than the Swiss rates reported by de Quervain et al. [63], who found rates of up to 18.4%. This discrepancy could be attributed to the high socioeconomic status of our sample, which might have offered some protective factors against severe depressive symptoms. The association between parental psychopathology and child psychopathology can be explained by several factors: 1) increased parent-child conflicts: Research by Kane and Garber [64] has shown that parental depression can lead to heightened parent-child conflicts, which in turn exacerbate behavioral problems in children. 2) Genetic factors: Hetterma et al. [65] highlighted the role of genetic predispositions in the transmission of psychopathology from parents to children. 3) Pandemic-specific stressors: During the pandemic, parents experiencing higher levels of anxiety and depression also reported increased parenting stress. This stress can negatively impact children’s behaviors [60]. 4) Parental support role: Children, particularly during stressful events, depend significantly on their parents/caregivers to help them process these experiences due to their underdeveloped cognitive and language skills. When parents are struggling with mental health issues, their ability to provide this crucial support can be impaired [66].

Finally, this study also examined the role of child worries (i.e., negative pandemic-related appraisals) in predicting trajectories of child behavior. Notably, parent-reported child worries emerged as a significant predictor for anxiety and affective symptoms in the young children. Children exhibited more worries about others than about themselves, indicating a pronounced concern for the well-being of others during the pandemic. These findings are consistent with previous research on older children and adolescents, which has demonstrated strong associations between various appraisals and internalizing symptoms during the COVID-19 pandemic [67–70]. Moreover, our results align with Vasileva et al. [10], who identified heightened concerns about personal and others’ health among young children affected by the pandemic and suggested a connection between these cognitions and subsequent behavioral changes. The limited cognitive skills of young children might make it more difficult for them to understand the pandemic and the control measures, leading to inappropriate worries. Further corroborating our findings, Murray et al. [22] reported that child worries specifically about COVID-19 were associated with increased emotional problems. However, the direction of this association currently remains unclear, warranting further investigation to determine whether heightened child worries lead to increased emotional issues or if preexisting emotional problems exacerbate worries about the pandemic.

### Strengths and limitations

One of the primary strengths of this study is its longitudinal design. This is the first study examining different trajectory classes in a sample of very young children. This is particularly noteworthy given that the effects of stressors and the impact of COVID-19 measures on daily life are highly age-dependent [6,71]. Focusing on infants and preschool children allows for differentiated statements about the pandemic’s impact on this specific age group, which is highly needed. Furthermore, the considerable sample size enables valid conclusions about the observed correlations. All of the measurement instruments used (CBCL 11/2 - 5, PHQ-9, GAD-7) are well-established and well-validated in research, facilitating comparisons with other studies.

However, there are also some limitations to our study. First, we did not collect any pre-pandemic baseline data. It is therefore unclear whether the individuals in the higher symptom trajectories had behavioral or emotional problems before the pandemic. This limitation applies to most studies, due to the unforeseeable emergence of the COVID-19 pandemic. We did, however, start collecting data already in April 2020, marking the very start of growing reported cases and governmental measures [72] which allows us to report very early reactions of young children. Second, we registered a significant number of dropouts, especially between the first two measurement instances. However, we imputed missing data and checked for differences between participants and non-participants based on the available T1-data to account for this limitation. The lower participation rates of parents with higher levels of depression and anxiety in the T2 and T3 assessments, as well as the underrepresentation of parents with lower education in the fourth assessment, may have introduced a bias that skewed our findings in an overly positive direction. Third, our sample is a convenience sample, meaning the findings cannot be directly generalized to the entire population of German-speaking countries and there was an observable trend towards a higher educational level. Lastly, parental reports were used to measure child behavior and appraisals which could prompt biased reporting, in particular regarding the association between parental and child mental health. This could have influenced our results because psychologically distressed parents may perceive their children as more burdened and attribute more negative appraisals to them. Moreover, internalizing behavioral problems are harder to observe than externalizing problems. This could have led to increased reporting of externalizing behaviors. A final limitation of this study is that the vast majority of participants were mothers which limits the generalization of findings to fathers (specifically with regard to parental mental health).

### Implications

Our findings have several clinical implications. First, the identification of a relatively stable group of young children exhibiting high levels of symptoms across all assessments underscores the importance of early detection and targeted interventions. Second, our results highlight the importance of considering child sex as a significant factor in the trajectory of mental health problems among preschoolers. While girls are more prone to developing affective symptoms, boys tend to exhibit higher levels of oppositional-defiant behavior. These differences underscore the need for tailored interventions that address the specific mental health needs of boys and girls in the context of pandemic-related stressors. Third, young children’s worries related to COVID-19 should be regularly assessed as they play a crucial role for behavioral and emotional problems. Dysfunctional child cognitions could be corrected through parent-child discussions about the stressor, which has been shown to reduce psychopathological symptoms in older children [10,73]. Fourth, since parental mental health significantly affects the behavior of preschoolers, likely through altered parental availability and altered parenting practices, interventions should focus on both psychological support of affected parents and improvement of parenting practices. As highlighted by Fosco et al. [24], interventions should enhance family cohesion and management of conflicts.

Future research should continue to explore the sex differences observed in our study and examine underlying mechanisms to develop effective strategies for supporting children’s mental health during and after pandemics. Comprehensive literature reviews and meta-analyses are needed for infant and preschool age groups to consolidate current findings and identify gaps in the literature. The presence of a chronically problematic class in all assessed problem domains suggests that chronic mental and behavioral problems in children during the pandemic should be monitored over the long-term [74]. At present it is unclear, how these problems develop over longer time. Studies on COVID-19 impacts require population-representative samples to ensure generalizability. Additionally, the promising results of cognitive assessments in young children suggest that these tools should be utilized more in research. By regularly assessing young children’s cognitions related to stressful life-events, researchers can better understand the impact of the pandemic on mental health. A comprehensive research approach that includes longitudinal studies and diverse samples is essential for developing tailored interventions to support the mental health of young children during and after pandemics.

## Conclusion

In sum, this study has found significant impacts of the pandemic on emotional and behavioral problems of young children under the age of 6 years. A substantial proportion of children displayed clinically relevant symptoms over the first year of the pandemic, primarily predicted by parental mental health and parent-reported child worries. Moreover, sex differences in symptomatology were observed warranting further investigation. The results of this study highlight the vulnerability of this age group and underscore the need for specific support for parents and families. Additionally, age-appropriate means to explain the disease and the measures taken to combat it are crucial for helping young children understand the pandemic in an appropriate manner.

## Supporting information

S1 Table Statistical figures for anxiety, affective, and oppositional-defiant symptom class trajectories. (PDF)

S1 Data Datafile. (CSV)

S1 Codebook Codebook for datafile. (XLSX)
