# Supplementary material for: Trajectories of emotional and behavioral problems in young children during the COVID-19 pandemic: A longitudinal study
Source: PLOS Ment Health. 2025 Feb 27;2(2):e0000265. doi: 10.1371/journal.pmen.0000265 (PMC12798200; doi:10.1371/journal.pmen.0000265)
Supplement: S1 Table — (PDF) [file pmen.0000265.s001.pdf]

		**Anxiety**								
		Intercept			Slope			Quadratic Term		
C	*n*	Est.	*SE*	*p*	Est.	*SE*	*p*	Est.	*SE*	*p*
1	761	2.24	0.11	**<.001**	-0.22	0.12	.07	0.09	0.04	**.03**
2	139	6.47	0.38	**<.001**	1.18	0.67	.08	-0.27	0.22	.23
		**Affective**								
		Intercept			Slope			Quadratic Term		
C	*n*	Est.	*SE*	*p*	Est.	*SE*	*p*	Est.	*SE*	*p*
1	770	1.68	0.10	**<.001**	-0.22	0.10	**.03**	0.09	0.03	**.006**
2	130	6.50	0.49	**<.001**	-0.90	0.49	.07	0.33	0.15	**.03**
		**Oppositional-defiant**								
		Intercept			Slope			Quadratic Term		
C	*n*	Est.	*SE*	*p*	Est.	*SE*	*p*	Est.	*SE*	*p*
1	343	1.83	0.16	**<.001**	0.06	0.17	.74	0.002	0.05	.98
2	447	4.93	0.30	**<.001**	-0.16	0.21	.43	0.07	0.06	.28
3	110	8.53	0.42	**<.001**	-0.63	0.52	.23	0.26	0.16	.12

C = Class (for anxiety and affective: 1 = low-symptom class, 2 = high-symptom class; for oppositional defiant: 1 = low-symptom class, 2 = medium-symptom class, 3 = high-symptom class); Est. = Estimate.
